# An infrastructure for qualified data sharing and team science in late-stage translational spinal cord injury research

**DOI:** 10.1016/j.expneurol.2024.114995

**Published:** 2024-10-10

**Authors:** J. Russell Huie, Abel Torres-Espin, Jeffrey Sacramento, Anastasia V. Keller, Wilsaan M. Joiner, Ryan North, David J. Reinkensmeyer, Ephron S. Rosenzweig, Jacob Koffler, Mark H. Tuszynski, Carolyn J. Sparrey, Jessica L. Nielson, Michael S. Beattie, Jacqueline C. Bresnahan, Jeffrey S. Grethe, Adam R. Ferguson

**Affiliations:** a Brain and Spinal Injury Center (BASIC), Department of Neurological Surgery, Weill Institute for Neurosciences, University of California, San Francisco, USA; b San Francisco Veterans Affairs Health Care System, USA; c Department of Physical Therapy, Faculty of Rehabilitation Medicine, University of Alberta, Edmonton, AB, Canada; d School of Public Health Sciences, University of Waterloo, ON, Canada; e University of California, Davis, CA, USA; f University of California, Irvine, CA, USA; g University of California, San Diego, CA, USA; h San Diego Veterans Affairs Health Care System, USA; i Simon Fraser University, Vancouver, BC, Canada; j Department of Psychiatry & Behavioral Sciences, University of Minnesota, Minneapolis, MN, USA

**Keywords:** Data sharing, Spinal cord injury, Informatics, Data science, Scientific data repositories

## Abstract

The complex and heterogeneous nature of spinal cord injury has limited translational bench-to-bedside results. The wide variety of data, including injury parameters, biochemical, histological, and behavioral outcome measures represent a ‘big data’ problem, calling for modern data science solutions. There are some instances in which SCI researchers collect sensitive data that needs to remain private, such as datasets designed to meet regulatory approval, sensitive intellectual property, and non-human primate studies. For these types of data, we have developed a Private Data Commons for SCI (PDC-SCI). Our objective is to give an overview of this novel data commons, describing how this type of commons works, how it can benefit the research community, and the cases in which it would be most useful. This private infrastructure is ideal for multi-lab transdisciplinary studies that require a well-organized, scalable data commons for rapid data sharing within a closed, distributed team. As a use-case for the PDC-SCI, we demonstrate the VA Gordon Mansfield SCI Consortium, in which multimodal data from behavior, biomechanics of injury, hospital records, imaging, and histology are integrated, shared, and analyzed to facilitate insights and knowledge discovery.

## 1. Introduction

As research continues to expand beyond the walls of a single lab, the need for cloud-based data management for team science has never been greater. The COVID-19 pandemic has shown that while generating data remotely can be done, the work of organizing, sharing, and analyzing data from disparate environments remains challenging (Alhomdy et al., 2021). In clinical informatics, cloud-based solutions for human-subjects research such as the Research Electronic Data Capture (REDCap) enable large scale sharing of targeted clinical endpoints (Harris et al., 2009). In addition, the NIH has long supported data-sharing in genomics through the gene expression omnibus (GEO) and the DataBase of Genotypes and Phenotypes (DBGaP) (Edgar et al., 2002; Mailman et al., 2007). However, multisite preclinical and translational projects require unique data solutions that do not fit neatly into these existing biomedical informatics infrastructures.

For example, late-stage translational development of regenerative therapies for spinal cord injury (SCI) requires researchers to manage data collected across multiple geographical regions, as well as across different areas of expertise. This type of research is characterized by unique data elements reflecting novel biological measurements including robotic rehabilitation devices (Fong et al., 2005; Reinkensmeyer et al., 2006), stem cell technologies (Rosenzweig et al., 2018), tissue scaffold engineering (Koffler et al., 2019; Yousefifard et al., 2020) and neuromodulation device data (Capogrosso et al., 2016; Wenger et al., 2016). These heterogenous data represent ‘big data’ characterized by the 3 V’s of high data *volume*, rapid data *velocity*, and wide data *variety* (Huie et al., 2018). The problem of high data volume is often associated with imaging and large clinical trials research where there is a single primary domain of study and the goal is to acquire high number of subjects (high ‘n’) to achieve a pre-defined level of statistical power on the primary outcome metric. In contrast, late-stage discovery and translational research typically involves a small number of large-animal subjects studied in great detail on numerous endpoints, and the problem becomes managing wide data *variety* (Huie et al., 2018). This is evident in stem cell biology and neuromodulation projects for neurological trauma, where data collected on a single subject span molecular and cellular endpoints, morphology, neurophysiology, behavior, and heath records. Managing such data, sharing them among team members, and making them interoperable with advanced analytics such as machine learning and artificial intelligence tools remain central challenges.

To facilitate data sharing within and between labs, we have previously developed the Open Data Commons for Spinal Cord Injury (odc-sci.org (Fouad et al., 2020; Torres-Espín et al., 2022) and the Open Data Commons for Traumatic Brain Injury (odc-tbi.org (Chou et al., 2021), which provide mechanisms for data management, open data publishing, and data citation. However, there are instances in which researchers collect sensitive data that needs to remain private, such as datasets designed to meet regulatory approval (e.g., FDA), sensitive intellectual property, and non-human primate studies, among others. To meet these challenges, we have created a private data commons (PDC) and deployed it for spinal cord injury research as a first use-case. The PDC is a cloud-based infrastructure that allows a team to store, manage, and share data from a wide variety of data types, where each type of data is generated at separate sites. The PDC acts as a central repository for all data collected and is designed to facilitate sharing and data integration within and across member sites that make up a team as a whole. The goal of this paper is to give an overview of this novel domain specific data commons for the SCI field, describing how this type of commons works, how it can benefit the research community, and the cases in which it would be most useful.

## 2. Methods

**Fig. 1.**
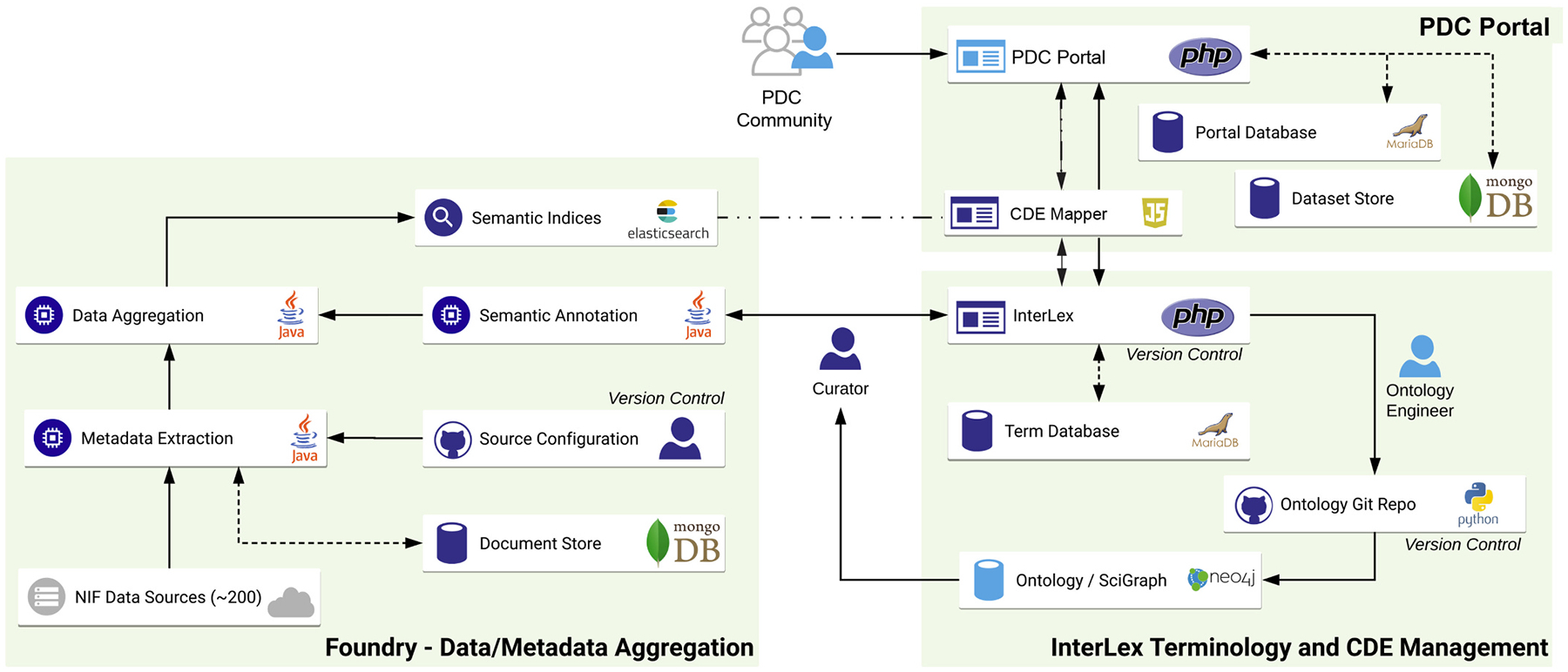
Private Data Commons Infrastructure. The private data software architecture is comprised of three main modules: Foundry, for Data Aggregation, metadata extraction, and version control; Terminology and CDE management using, for building and controlling ontologies; and the PDC portal, where other modules are integrated, datasets are stored, and the secure user-facing interface can be used by those with qualified access.

The Private Data Commons is built upon software architecture previously developed for the Open Data Commons for spinal cord injury (odc-sci.org), the NIH-supported, domain specific repository for the field of of SCI (Repositories for Sharing Scientific Data | Data Sharing, 2024; Torres-Espín et al., 2022). The repository infrastructure, known as the FAIR Data Informatics (FDI) Commons infrastructure, is built on top of the SciCrunch cyberinfrastructure housed in the Cal-IT2 datacenter and the San Diego Supercomputer Center at the University of California, San Diego. As with odc-sci.org, the private data commons leverages e-commerce grade encryption, full data provenance tracking and change logging, offsite backup and data recovery among other features to ensure data security and integrity. The FDI Commons infrastructure, is built in 3 modules (Fig. 1): The Private Data Commons Portal, which serves the end user and can host analytical applications via application programming interfaces (APIs); Interlex, which manages terminologies and common data elements for use by the Portal and APIs; and Foundry, which aggregates information from external data resources and enables integration with these resources.

## 3. Results

### 3.1. Resource infrastructure and governance

The private data commons for spinal cord injury (PDC-SCI) addresses the data management needs for multisite team science, in a *private* repository. The need for privacy arises in research cases such as: data pending FDA approval, safeguarding intellectual property, or sensitive data including non-human primate research. Data sharing is facilitated by proper data *governance*, and the principles of proper data governance should be applied not only when sharing data publicly to the broader community, but also *within* a multisite, team science project. To this end, the PDC has been designed to adhere to the FAIR principles of data governance (Wilkinson et al., 2016). Briefly, FAIR states that data must be Findable, Accessible, Interoperable, and Reusable. First, data must be findable. That means data and metadata need to be sufficiently annotated and tagged such that it can be searchable, commonly by using search algorithms. Data then needs to be accessible; users should be able to visualize and download the data, ideally in a standardized format that users can retrieve easily. Third, data need to be interoperable. This means that data are structured in a way that allows it to be aggregated with other datasets, operated on using different computer algorithms, easily readable by both humans and computers, and must also be in a format that allows for use with other programs, such as analytical software. Finally, data must be reusable. This is ultimately the goal of data sharing, to allow for users to combine datasets and create new analyses that are reproducible. This requires that metadata are clear and concise so that users are fully informed as to what the data represent.

### 3.2. Use case: veterans affairs gordon mansfield spinal cord injury consortium

**Fig. 2.**
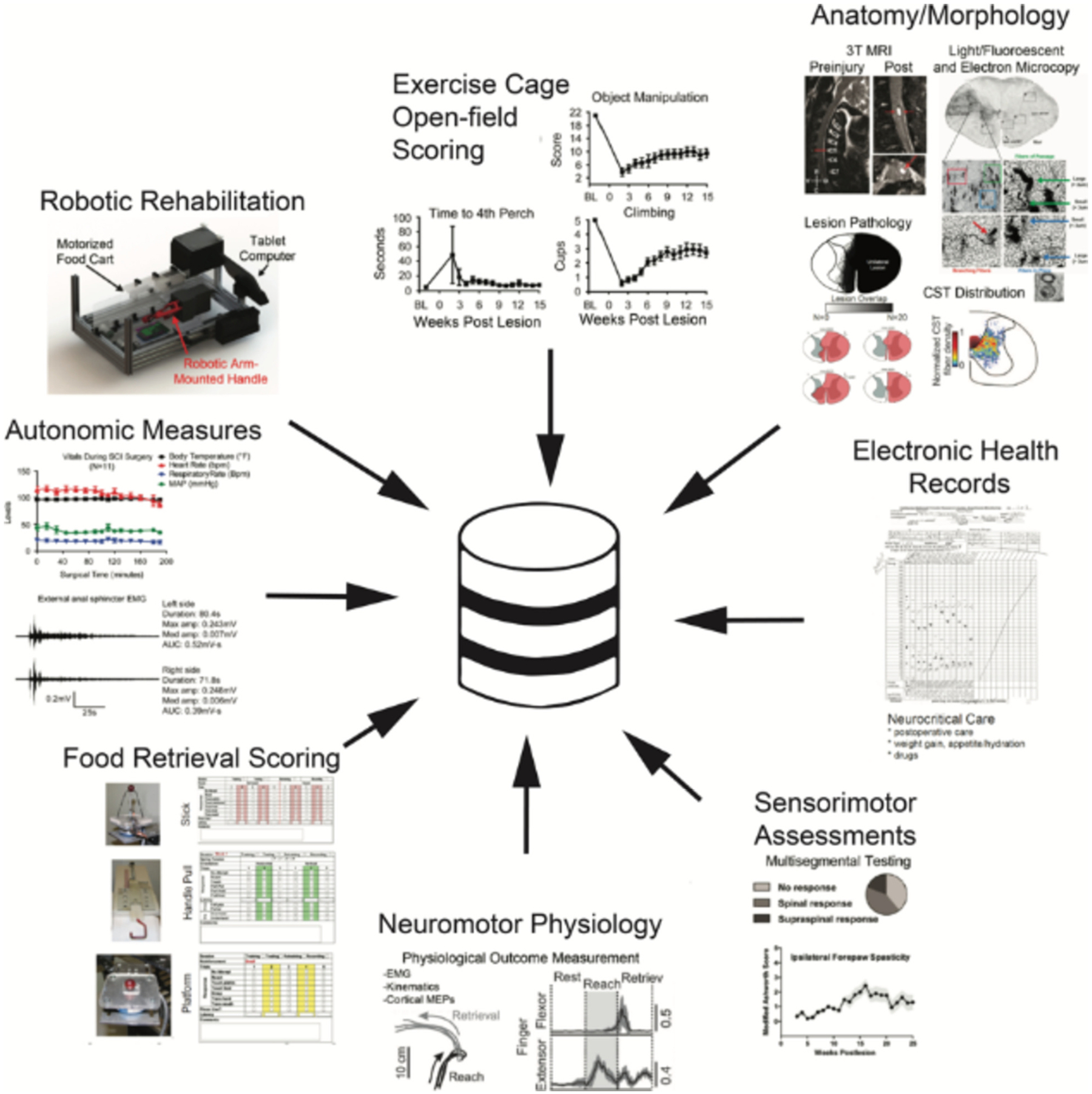
Multimodal Data Ingested to a Private Data Commons for Team Science. This figure illustrates the various types of data collected in the VA Gordon Mansfield Consortium for Spinal Cord Injury. This project spans a number of universities, each with a specific focus/ particular aspects of spinal cord injury experiment. The PDC-SCI houses these disparate modalities in a central repository that allows for easy access within and across labs in the consortium, but is kept private and secure, only to be used by those with access clearance.

The PDC-SCI has been a crucial organizing element behind one of the most sophisticated and complex late-stage translational spinal cord injury research projects: the Gordon Mansfield Spinal Cord Injury Consortium (VAGMSCI). This team science project consists of multiple labs across 5 University of California campuses (UC San Diego, UC Irvine, UC Los Angeles, UC San Francisco, and UC Davis). The VAGMSCI is a transdisciplinary effort to test cutting-edge therapies for spinal cord injury in non-human primates. The group collects a wealth of data across a number of research domains, including: complete veterinary health records, highly granular time series data on the biomechanics of injury, a battery of behavioral outcomes, physiological measures, pre- and post-injury MR imaging, and histological outcome measures, including axonal sprouting, stem cell survival/proliferation, and detailed connectome data (Fig. 2). Each of these data domains is managed at separate universities, and the PDC is organized into separate lab spaces for each of these units. As new data is generated, organized, and analyzed, the data are pushed to the PDC and housed within these labs. While each separate domain can go from raw data collection to analysis and final publishable results, it is the sharing of data across labs that is crucial to the mission of the consortium, and it is the integration of these multimodal data that will generate unique knowledge discovery. Thus, a major tenet of the PDC is that although this is a private community, data sharing is guided by the same FAIR governance principles that undergird public data sharing. In this way, the PDC acts as a microcosm of the broader neurotrauma community.

**Fig. 3.**
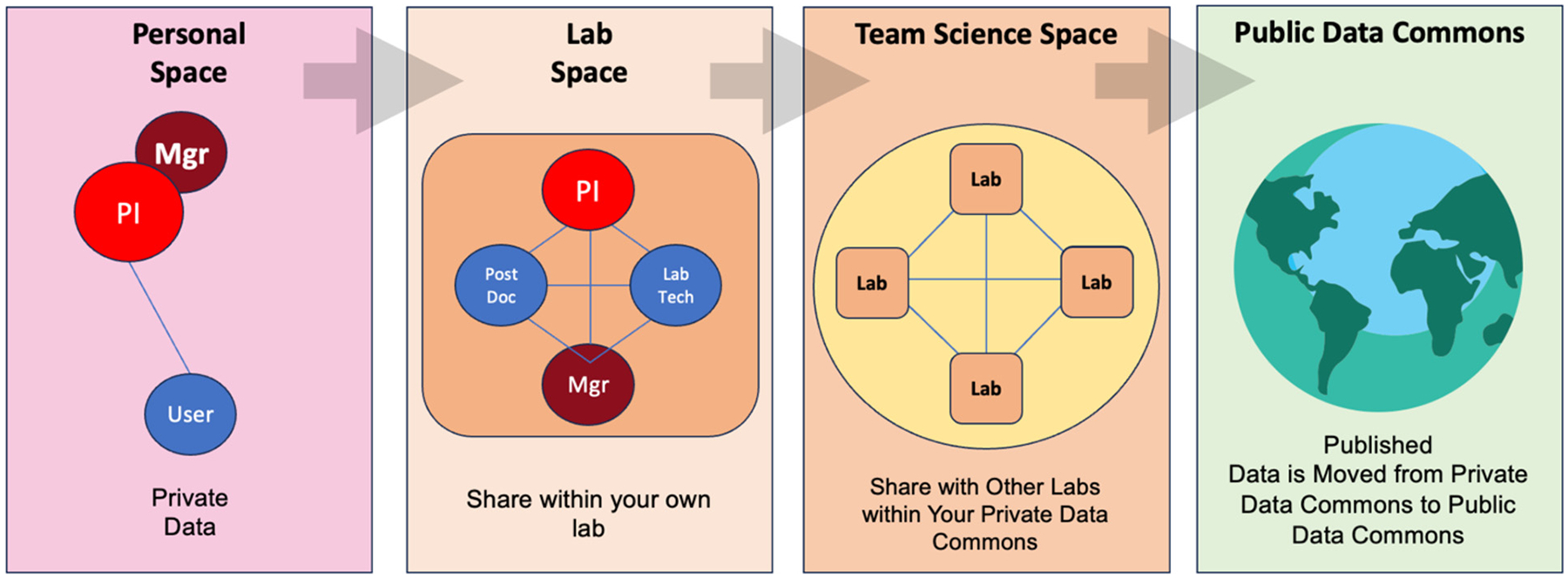
Movement of Data through Spaces, form Personal to Published. The private data commons is designed so that the principal investigator (PI) has complete control of the access that one has to the data at all times. Data can begin in a private space, that is only accessed by the PI and/or lab manager, and those to whom the PI or a PI-designated lab manager (Mgr) gives access. The data can then be moved to lab space, where all vetted members of a particular lab have access. In the private data commons, there is also the option to share with other labs that are working on the same overarching team science project. Finally, when data are ready to be published, the dataset and all accompanying metadata are moved to the public data commons where data can be found, accessed, and reused in accordance with the FAIR principles of data governance.

The SFVAMGC PDC currently houses seven distinct labs that represent the different data domains (Veterinary Health Records, Demographics, Injury Biomechanics, Imaging, Biofluid Biomarkers, Behavior, Robotic Rehabilitation Training, and Informatics/Analytics). As of June 2024, a total of 169 datasets have been collected and uploaded across these virtual labs. Each lab is headed by a principal investigator (PI) from the team and is accessed by lab members that have been approved and verified. Any of these lab members have the authority to upload data into their respective labs. As a dataset is uploaded, the lab member and PI work together to decide the extent to which the data can be accessed by others, moving from most private to fully public ‘spaces’. When a dataset is first uploaded it is initially accessible only to the uploader (the ‘Personal Space’). When data are ready to be shared within the lab, it can then be moved by the lab member to the ‘Lab Space’. From here, any lab member, manager, or PI within the lab can access and update the dataset. When labs are prepared to integrate and share datasets, the data can then be virtually moved to the ‘Community Space’. At this point anyone with user permission to access the private data commons can now access and interact with the dataset (Fig. 3).

### 3.3. Data publishing

The open sharing of the data that underlie published works is a growing mandate in scientific research. The National Institutes of Health now require that all data collected with NIH funding be made public as the results of these data are published (Final NIH Policy for Data Management and Sharing, 2024). Given this mandate, the PDC was created with the ultimate goal of dataset publishing in mind. When your scientific journal article is published, the underlying dataset that was used to generate figures and results will be published as well through a partnership with the Open Data Commons for SCI (odc-sci.org), the NIH-supported repository for spinal cord injury data management and sharing. Data creators work closely with the PDC editors to ensure that the dataset meets minimum FAIR standards. This package, that includes the dataset, data dictionary, study metadata, and provenance, is then minted a digital object identifier (DOI), which is linked to this information in perpetuity. Much like a copyrighted published journal, a published dataset can be reused under a Creative Commons Attribution (CC-BY) open license (https://creativecommons.org/licenses/by/4.0/?ref=chooser-v1), which allows for anyone to reuse the dataset as long it is cited. The citation allows the dataset creators to receive credit for their work, and just as with journal authorship, citation of a dataset will be recognized as scientific contribution (e.g., driving metrics such as H-index) of all listed contributors to a dataset, as long as the data citation is listed in the references of a publication.

As an example, we include here the underlying raw data from a seminal work by Rosenzweig et al. in 2019 (20). This study was designed to test the effects of an enzyme (chondroitinase) that was hypothesized to have beneficial effects after spinal cord injury. The dataset consists of functional recovery tasks measured over time, as well as corticospinal tracing data that measured changes in axon and synaptic density in response to chondroitinase treatment.

**Fig. 4.**
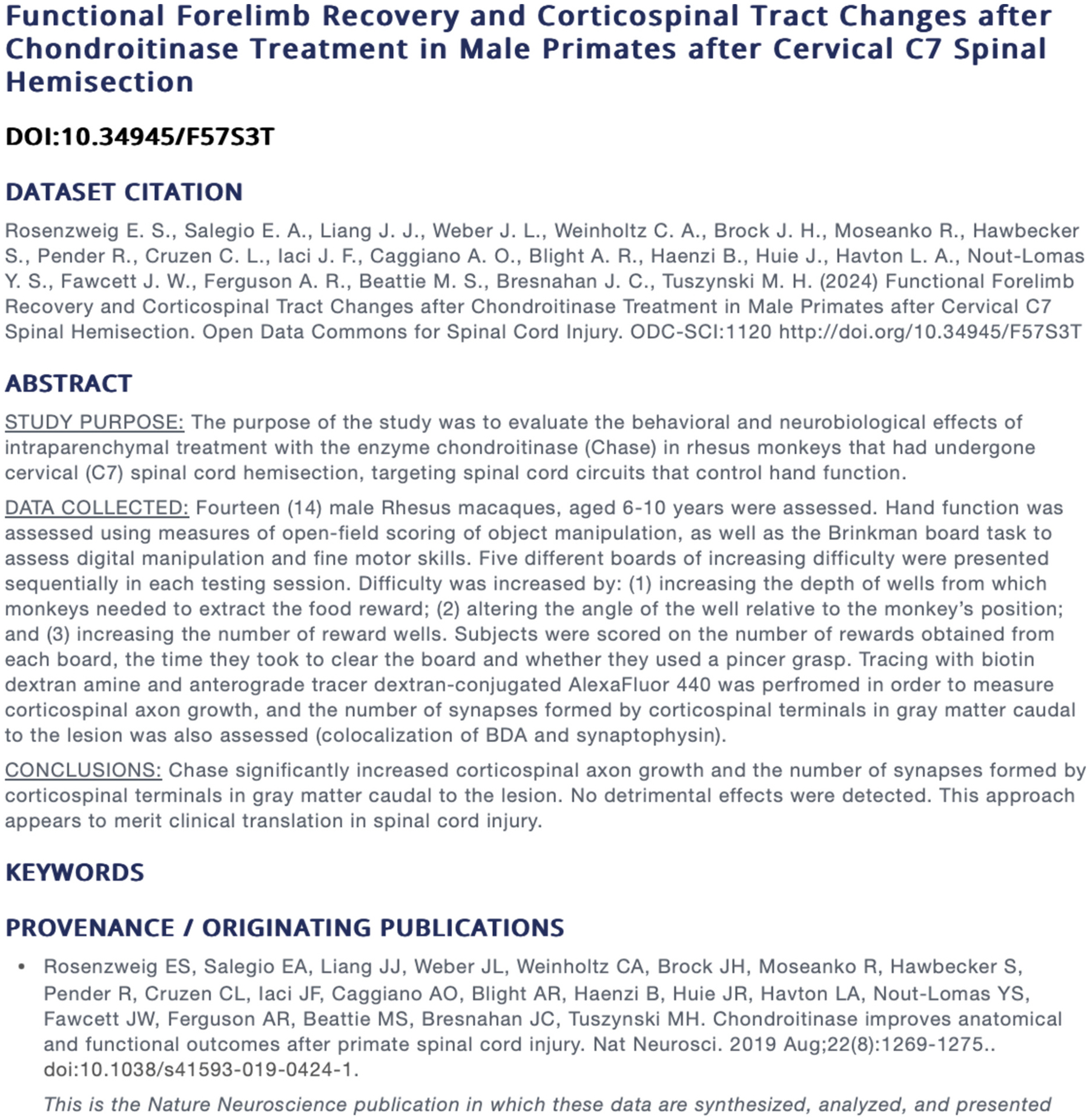
Metadata for Published Dataset. This screengrab illustrates the study metadata that accompanies a particular published dataset. The dataset citation looks similar to a journal article citation, and is cited in the same way. The abstract details all of the specifics that one needs in order to know the type of data that will be found within the dataset. Provenance also shows the journal articles in which these data have been presented. https://doi.org/10.34945/F57S3T.

These data were collected and stored in the PDC throughout the course of the study, and were then formatted for publication, at which time a DOI was requested. The metadata (see Fig. 4) and data dictionary were then reviewed by independent data analysts to check that 1) the metadata were sufficiently descriptive for future use, 2) the data dictionary accurately and thoroughly represented and described the variables that were included in the dataset. This quality assurance process is important to make the data reusable, by ensuring that all aspects of the data that were collected are clear and concise.

The dataset is publically accessible under a persistent digital object identifier (DOI) at https://doi.org/10.34945/F57S3T.

### 3.4. Interoperability with multidimensional analysis

**Fig. 5.**
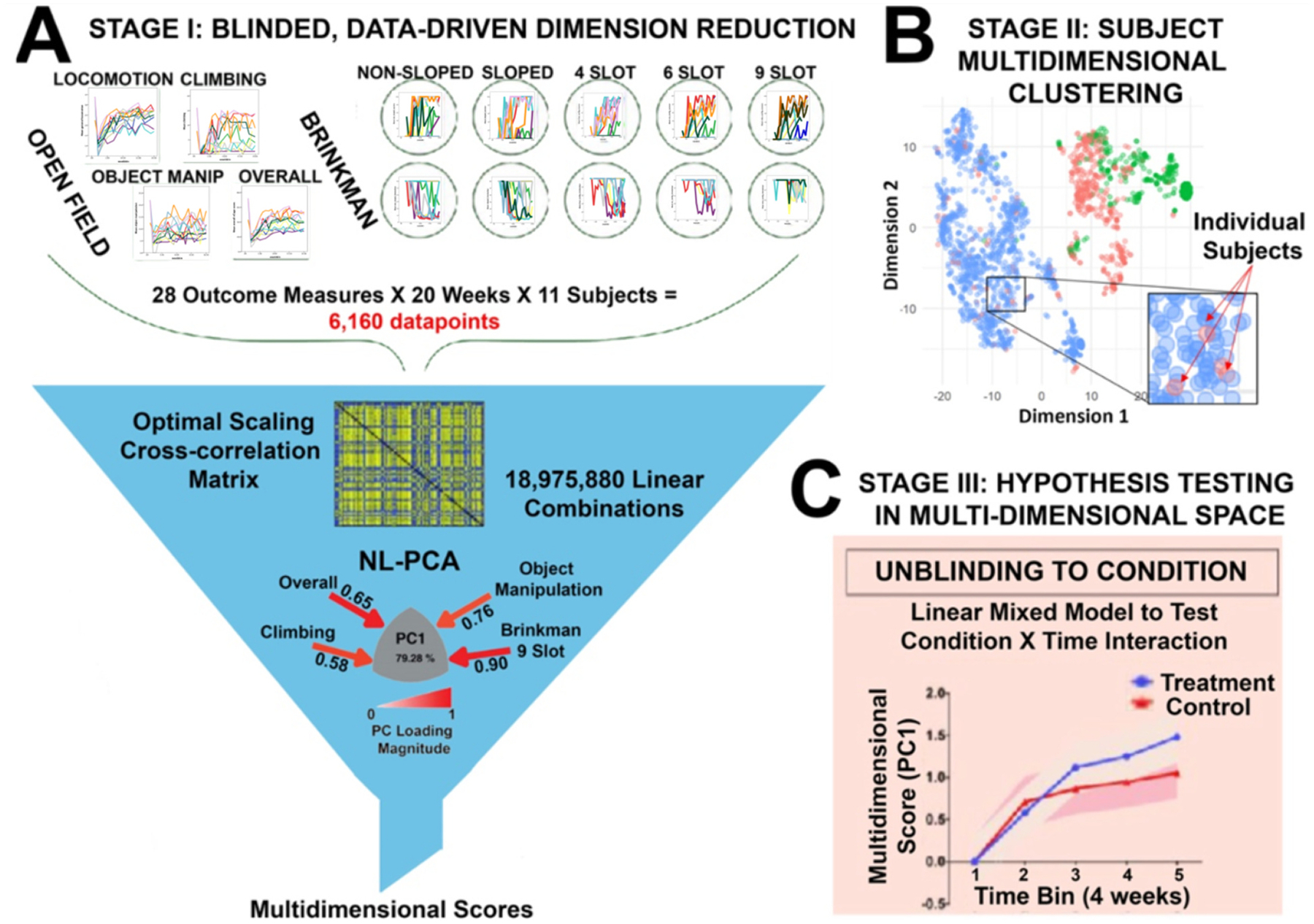
Integrating transdisciplinary data for analytics/knowledge discovery. When multimodal data can be easily accessed, big data analytic tools can help to drive knowledge discovery. In this example (A), the different forms of data are combined in a blinded, data-driven way, such as principal components analysis. B, other techniques such as multidimensional clustering, can help researchers better understand how groups of subjects are similar or different, in order to identify patterns that may be otherwise difficult to detect. C, after dimensionality reduction, a single score that reflects a subject’s place in the multidimensional (or *syndromic*) space can be used for hypothesis testing. This process avoids the analytical pitfalls that are common if one attempts to run multiple hypothesis tests on a large group of univariate measures.

As our ability to collect and manage big data improves, so too does the need for analytics that leverage the complexity of these data. The analytics core of the VAGMSCI has worked over the past decade to advocate the idea of *syndromic* analyses, in which a multi-modal, multidimensional, integrated approach to knowledge discovery is favored over running large numbers of univariate analyses (Ferguson et al., 2011; Torres-Espín et al., 2021). The multimodal nature of this consortium is well-suited for multivariate analytics, and the PDC is designed to facilitate these analyses. In the past 8 years, a number of research articles have been published by this consortium that take advantage of the opportunity for data integration to drive multidimensional analytical workflows across multimodal data including medical imaging, electrophysiology, veterinary medical records, multi-omics, neurobehavioral assessments, neuromodulation technology development, and neuromorphology and pathoanatomy after SCI (Brock et al., 2018; Kumamaru et al., 2018; Nielson et al., 2015; Rosenzweig et al., 2019; Rosenzweig et al., 2018) (Fig. 5). The combination of high volume and wide variety allows for a first stage data-driven dimension reduction. For example, combining disparate open field locomotion and object manipulation with tests of finer motor control created a correlation matrix consisting of over 18 million linear combinations. This high dimensionality was then reduced using principal component analysis to produce single composite scores for each individual that represented the unique pattern of interaction between these tasks that accounted for nearly 80 % of the variance in the data (Fig. 5A). In stage 2, individual subjects can be plotted based on these multidimensional scores, where methods such as topological data analysis can then be used to identify unique clusters of individuals (Fig. 5B). Finally, in stage 3, only after the complexity and variability across outcome measures and individuals have been modeled and accounted for, will hypothesis testing be performed (Fig. 5C). This workflow, made possible by close and careful data management within the PDC, has the power to detect effects that may have been lost or diluted by the initial variety of outcome measures, and perhaps more importantly, avoids the possible pitfalls of multiple univariate comparisons that are a major driver of the current reproducibility crisis (Haefeli et al., 2017; Ioannidis, 2005)

## 4. Discussion

From the outset, the PDC was designed with the importance of proper data governance in mind. Much like an open data commons, the PDC is dedicated to ensuring that the sharing and ownership of data, as well as data provenance, are transparent and legally sound. Clear and concise documentation, including Data Use Agreements and Memoranda of Understanding, is foundational to our data management and data sharing efforts.

Any data commons that is designed to intake sensitive data, such as de-identified human patient data, must navigate the regulatory hurdles that are in place to ensure that privacy is maintained. But there are a number of burgeoning efforts and tools that are helping researchers to ensure de-identification. Tools such as the Protected Health Information Filter (or *PHIlter*) a software algorithm that scans free-text clinical notes and removes identifying information, are making the de-identification process easier and clearer (Norgeot et al., 2020). Similarly, the NIH has introduced the NLM Scrubber, a tool that can also scan electronic health records to make them de-identified, and other similar tools have also been developed (Heider et al., 2020). These tools enable open data reuse for a wealth of big-data across all health fields for assessment and analysis, while maintaining patient privacy. While the PDC aims to help support researchers and educate them about data privacy issues, ultimately the responsibility for de-identification of data rests with the uploader. Users of the PDC must adhere to the data sharing rules that are put in place by the institutional review boards and privacy laws that govern their data stewardship. As more sensitive data becomes available and shared, both within consortia and the broader research community, there will be a continued need for better ways to monitor and assess these privacy issues. To that end the NIH Office of Data Science Strategy have created calls to further fund the development of tools to help in biomedical data stewardship these efforts (Home Page | Grants and Funding, 2024).

The PDC continues to grow and expand, not just in the number of datapoints it manages, but with new features as well. The PDC portal is equipped with API capability, which allows developers (including those with Python coding skills within the community) to build data visualization and analytical tools that can be used directly within the PDC. These new features will give researchers in the consortium the ability to monitor updates to the data in near real-time. For instance, as daily behavioral performance measures are uploaded to the PDC, PIs in the group will have the opportunity to generate time-series graphs and remain abreast of individual progress. This will also allow researchers to detect trends in the data that may help guide future healthcare decisions for the subjects. The PDC is also enabled with the capacity for two-factor authentication, to further secure access and verify users.

The PDC continues to develop best practices for the storage, management, and sharing of data types that are not quite as amenable to summarization in a tidy, flat spreadsheet as well. Current projects on this front include MR image analysis and storage, waveform data from injury biomechanics, and highly-granular neuromonitoring time-series data. Solving these data issues will provide even greater level of understanding of the nature of the evolving spinal cord injury.

## Data Availability

Data were deposited at the Open Data Commons for Spinal Cord Injury (ODC-SCI; RRID:SCR_016673). The ODC-SCI is a secure, cloud-based repository deigned to share research data. The dataset is publically accessible under a persistent digital object identifier (DOI) at https://doi.org/10.34945/F57S3T.
